# A Staged-OLIF approach can minimize construct lengths in adult spinal deformity- A case series and literature review

**DOI:** 10.37796/2211-8039.1648

**Published:** 2025-06-01

**Authors:** Guna pratheep Kalanchiam, Alexander Shao-Rong Pang, Jacob Yoong-Leong Oh

**Affiliations:** a Clinical Fellow, Spine Surgery Unit, Department of Orthopedics, Tan Tock Seng Hospital, Singapore; b Yong Loo Lin School of Medicine, National University of Singapore, Singapore; c Deputy Head of the Department of Orthopedics, Head of Service (Spine Surgery), Senior Consultant, Tan Tock Seng Hospital, Singapore

**Keywords:** Adult spinal deformity, Length of construct, Minimally invasive, OLIF, Staged approach

## Abstract

**Background:**

Adult Spinal Deformity (ASD) is one of the challenging conditions to treat for spine surgeons. One of the important dilemmas in the management of these patients is the decision on the number of levels of instrumentation and the overall length of the construct. OLIF has the advantage of providing a minimally invasive approach to address this complex pathology also allowing us to stage the procedure and thus help clinicians reassess if the patient requires an additional decompression, long fusion lengths, or need for osteotomies.

**Aim:**

Our study aims to evaluate the possibility of minimizing the construct length following a staged OLIF approach in ASD patients and also to analyze the clinical and radiological outcomes following a staged OLIF surgery.

**Methods:**

We present three cases of ASD, where the patients had a significant imbalance in either the coronal/sagittal profiles, and by using a staged approach, the surgeon was able to reduce the construct lengths and also operate on these deformities using an all Minimally Invasive Surgery (MIS) approach.

**Results:**

Overall sagittal and coronal profiles improved in all three cases with satisfactory fusion rates and VAS scores (back pain) post-operatively. Two of the three patients had an all-MIS approach (OLIF with MIS Ponte osteotomy and robotic guided pedicle screw instrumentation) and in one patient a four-rod construct was preferred to improve the stability. No complications were observed during a mean follow-up period of 4 years.

**Conclusion:**

A staged approach in ASD (lateral OLIF and posterior instrumentation) is a valuable surgical strategy for better correction of the coronal and sagittal plane deformities with relatively lesser construct length. It reduces the problems associated with prolonged anesthesia, in addition, it helps the surgeon in reassessing the pelvic parameters, thereby helping to decide on the need for additional osteotomies during the second procedure.

## 1. Introduction

Adult spinal deformity (ASD) is increasingly recognized as one of the most prevalent spinal conditions worldwide. This is primarily due to the increase in life expectancy of the aging population and improvements in the technical and technological aspects of spine care enabling early diagnosis and treatment [1]. However, it is one of the challenging conditions to treat due to the varied clinical presentation, physiological characteristics of the affected patient subgroup, the availability of different treatment options, and the lack of widely accepted standard treatment guidelines. Surgical management can often be coupled with a higher incidence of complications due to the presence of medical comorbidities in this patient subset, sarcopenia, osteoporosis, coronal and sagittal malalignment, need for longer levels of instrumentation, and the extensive soft tissue dissection [2,3]. All these factors emphasize the need for a Minimally invasive surgery approach with lesser concurrent damage to the normal anatomical structures.

## 2. Aim

Over the years, the posterior approach has been the main workhorse for the management of ASD. However, the degree of coronal correction achieved in these posterior-only techniques is relatively less compared to the anterior techniques. In spite of the better magnitude of deformity correction, the anterior approach has a significant surgical morbidity [4]. The lateral approach has gained attention in recent years due to its benefits of better deformity correction in both coronal and sagittal planes without traversing through the intra-peritoneal contents. It has emerged as a truly minimally invasive approach for adult spinal deformity. We report three cases of adult spinal deformity that were operated by a staged lateral and MIS/open posterior approach, their potential advantages and clinical outcomes.

## 3. Methods and surgical technique

Three cases of lumbar degenerative disc disease with adult spinal deformity treated at our hospital were used as illustrative cases. All the patients had a pre-operative coronal and/or sagittal imbalance and underwent a staged OLIF procedure. These patients had a minimum follow-up of 3 years. The study design complies with the Helsinki Declaration and informed consent was obtained from all patients. All three patients underwent a staged surgical approach. First Stage: two or more levels of Oblique Lumbar Interbody Fusion (OLIF), and the second stage involves posterior pedicle screw instrumentation (minimally invasive surgery (MIS)/open). The interval between the two stages was 3–5 days.

**Fig. 1:**
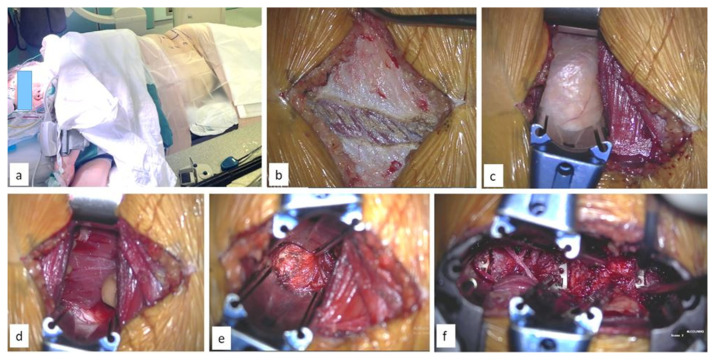
(a) showing the positioning of the patient in lateral decubitus position with skin markings at the lumbar region (b) skin incised and fascial incision over the external oblique musculature (c) showing retroperitoneal fat after retraction of the external oblique, internal oblique and transversus abdominis muscles (d) exposure of the disc space (e) placement of OLIF cages through the tubular retractors.

In the first stage, a left-sided retroperitoneal approach was used, and OLIF was performed with tubular retractors (Fig. 1). The decision regarding the fusion levels was based on the presence of instability/stenosis and the feasibility of the surgical corridor (anatomy of psoas musculature, location of the vascular structures). Intra-operatively, the disc space was thoroughly prepared under fluoroscopy guidance after cutting through the annulus. Shavers were used to remove the cartilaginous end plates and the opposite annulus was traversed using Cobb’s elevators. After trials of serial sizing, appropriately sized interbody cages were placed with Bone Morphogenic Protein (BMP-2) (Infuse, Medtronic, Memphis, TN, USA) or Demineralized Bone Matrix (DBM) (Grafton, Osteotech, Inc., Eatontown, NJ). Cages were placed in the middle of the disc space spanning along the ring apophysis of the vertebral body below. In cases requiring significant lordosis, the anterior cage position was preferred. All the steps were carefully performed using fluoroscopy guidance. Hemostasis was achieved using a hemostatic matrix (Floseal; Baxter Healthcare Corporation Fremont, CA 94555, USA), and a drain was placed when three or more levels were operated. Postoperatively, we performed supine lumbar spine and standing whole spine radiographs to assess the flexibility and the degree of correction of the deformity. The magnitude of coronal Cobb correction, pelvic parameters, sagittal vertical axis (SVA), and C7 plumb line were assessed. Based on these parameters, the need for additional procedures like posterior column osteotomies, and the extent of pedicle screw fixations were decided for the second stage.

**Fig. 2:**
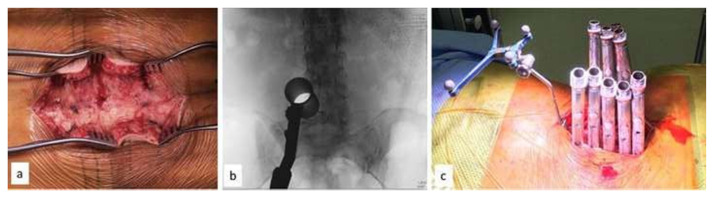
(a) Dorsolumbar fascia exposed bilaterally using a midline skin incision (b) docking of tubular retractor under fluoroscopy guidance for selective decompression of the lateral recess(c) showing placement of pedicle screws at appropriate levels using a minimally invasive approach. Note the Dynamic Reference Array (DRA) attached to the cranial spinous process for navigation.

In stage II, a posterior midline skin incision was performed, and using the MIS technique, pedicle screws were placed under robotic/navigation guidance (Fig. 2). Posterior column osteotomy (Ponte osteotomy) was performed at lumbar levels for correction of the deformity if needed. In cases necessitating fusion at L5–S1, transforaminal lumbar interbody fusion (TLIF) was performed. All these steps were performed using the MIS technique with an operating microscope and a tubular retractor system (Medtronic, Inc.). Deformity correction was performed using persuaders and reduction devices. In one of our patients, the standard open technique was adapted for posterior instrumentation. Postoperatively, patients were followed up at 1, 3, and 6 months, one year, and annually thereafter. Clinical and radiological evaluations were performed at every visit.

## 4. Results

### 4.1. Case examples

#### 4.1.1. Case 1

**Fig. 3:**
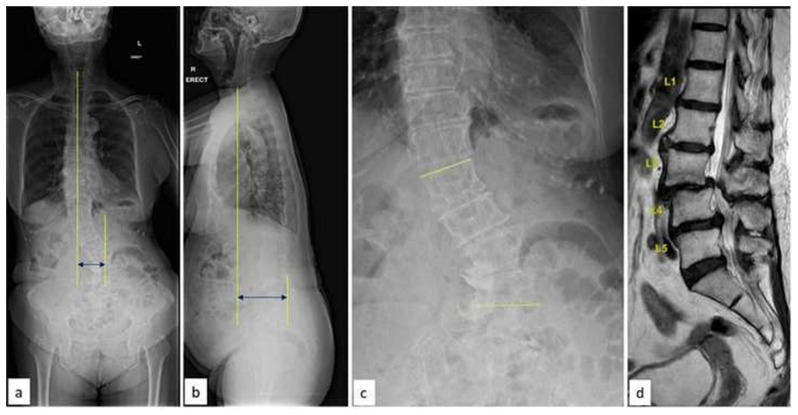
(a–b) Whole Spine Antero-Posterior and lateral radiographs showing coronal and sagittal imbalance in a patient with Degenerative lumbar scoliosis (c) AP radiograph of the lumbar spine showing the magnitude of the deformity (d) Mid-sagittal MRI showing L3–L4 anterolisthesis with multiple level disc degeneration.

**Fig. 4:**
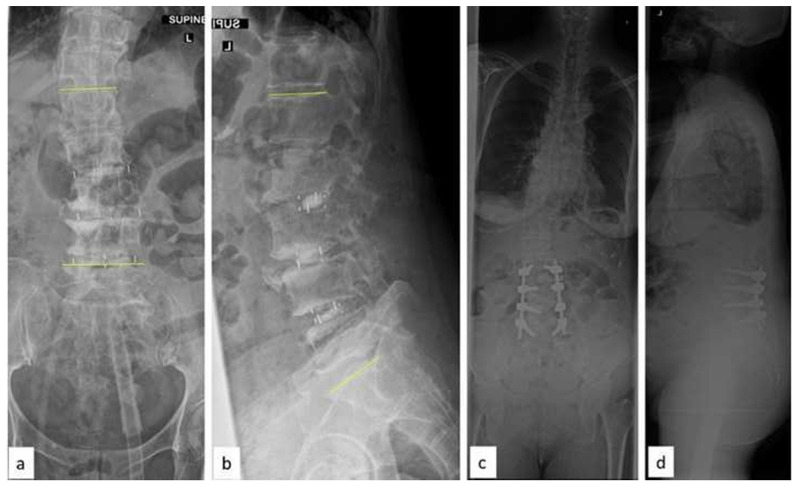
(a–b) Supine AP and Lateral radiographs after first-stage surgery. Note the correction of coronal Cobb’s and optimal lordosis using L2–L5 OLIF cages (c–d) Post-operative whole spine AP and Lateral radiographs after second stage surgery (L2–L5 MIS posterior instrumentation).

A 63-year-old female presented with low back pain (VAS: 7) and right gluteal pain radiating to the right lower limb for the past 2 years. She had a previous history of L5 selective nerve root blocks following failed conservative management. There was no significant improvement in symptoms and the patient had persistent neurogenic claudication. On clinical evaluation, the patient exhibited trunk listing to the left side. Neurologically, her motor power was normal (MRC grade 5/5) with reduced sensation from L2–L5 bilaterally. Radiographs showed degenerative lumbar scoliosis (right convex) and major curve apex at L2–3 with a lateral listhesis at the same level. L3–L4 and L4–L5 had grade I anterolisthesis and reduced lumbar lordosis (L1–S1) (33°). The magnitude of the primary curve (L2–L5) was 34.8°, she also had a positive sagittal imbalance (+8.5 cm), C7-CSVL was 5.2 cm and the pelvic parameters were PI: 57.6°, PT: 33.4°, SS: 16.4° (Fig. 3). Magnetic resonance imaging revealed significant stenosis at L3–L4 and L4–L5 levels. In the first stage, L2–L5 OLIF was performed. Postoperatively, the major curve cobb was reduced to 4.8°, and C7-CSVL was reduced to 2.8 cm. This is followed by L2–L5 percutaneous pedicle screw instrumentation with minimally invasive (MIS) tubular Ponte osteotomy from L2–L5 (Fig. 4). Postoperatively, lumbar lordosis improved to 45° and sagittal and coronal balance improved. At the final follow-up of three years, the patient was ambulant independently with no signs of claudication and with a follow-up VAS score (back pain) of 2.

#### 4.1.2. Case 2

**Fig. 5:**
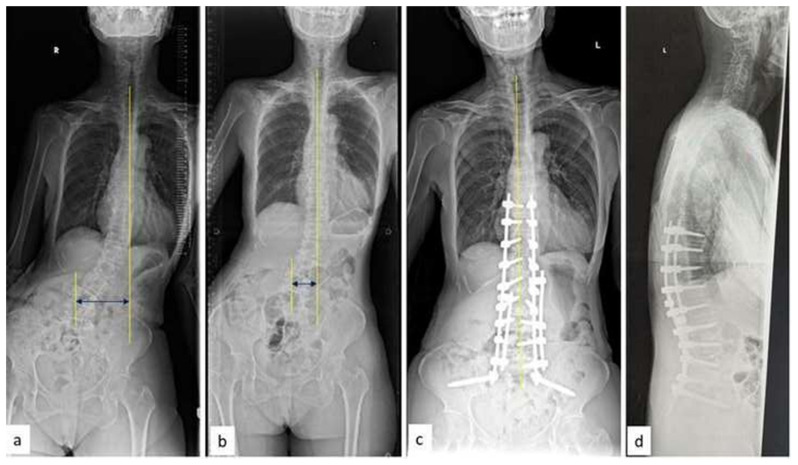
(a) Pre-operative Whole Spine Antero-Posterior radiograph showing lumbar scoliosis with significant imbalance in coronal axis (b) Whole Spine AP radiographs after stage I OLIF surgery showing about 70 % improvement in coronal balance (c–d) Three years follow-up radiographs (after T10-Pelvis posterior stabilization) showing a well-balanced spine in sagittal and coronal axes.

A 73-year-old female patient presented with chronic low back pain (VAS: 8) over 6 years, worsening for the past 12 years with bilateral anterior thigh pain. She was only community ambulant with the assistance of a walking aid due to neurologic claudication. She has a known history of systemic hypertension and hypothyroidism and is on regular treatment. Clinically, she had a significant listing of the trunk to the left and a rounded kyphosis of the thoracolumbar region. Neurological examination was normal and radiographs revealed a degenerative scoliosis of the lumbar spine (convex left) with an L5 transitional vertebra. There was a significant coronal imbalance and thoracolumbar kyphosis. The magnitude of the primary lumbar curve (D12–L3) was 33.4° and the fractional curve (L3–L5) was 16.9°. She had a severe coronal imbalance (CSVL-C7: 9.7 cm), and the pelvic parameters were PI: 44.7°, PT: 31.4°, SS: 8.1° (Fig. 5). She underwent L3–L5 OLIF as a stage I procedure, followed by L2–S1 Ponte osteotomy, and T10-pelvis posterior instrumentation with a four-rod construct in the second stage. Postoperatively, the major curve improved to 5.6°, lumbar lordosis was 35.6° (PI-LL: 9.1°) and physiological coronal balance was achieved (Fig. 5) and following surgery, the VAS score (back pain) improved to 1. At the final follow-up at three years, the correction was well-maintained without any evidence of implant failure.

#### 4.1.3. Case 3

**Fig. 6:**
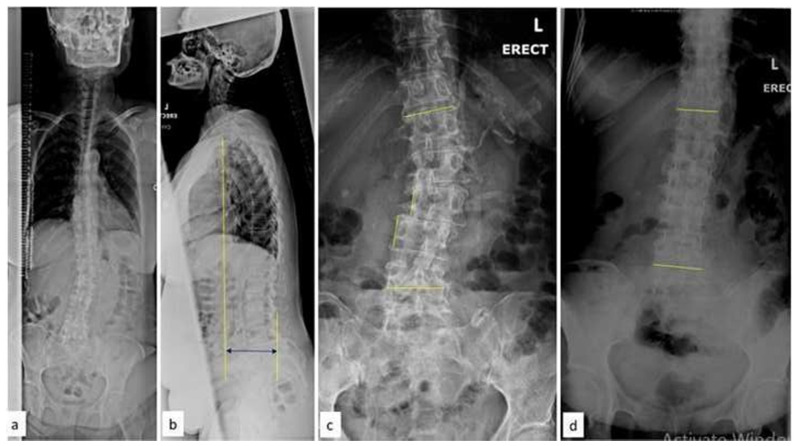
(a–b) Whole Spine AP and lateral radiographs with degenerative lumbar scoliosis. Note the CSVL anterior to the postero-superior aspect of S1 and the femoral heads (c) AP radiograph showing the magnitude of coronal deformity and lateral listhesis at L3–L4 level (d) AP radiograph of the lumbar spine after the first stage OLIF surgery. Note the significant correction of the deformity in coronal plane.

**Fig. 7:**
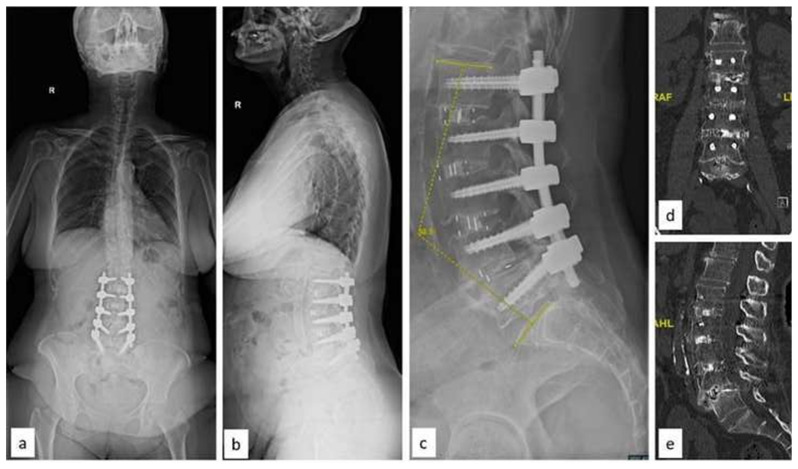
(a–b) Whole Spine AP and lateral radiographs following stage II: L4–L5 MIS TLIF with posterior pedicle screw fixation (c) Lateral lumbar radiograph showing correction of lumbar lordosis (L1–S1: 58.5°) (d–e) Four year follow-up CT scan showing solid fusion from L1–L5.

A 62-year-old female patient presented with left buttock pain, radiating to the left lower limb and associated numbness for six months. She also presented with neurogenic claudication and a progressive reduction in walking distance. Her comorbidities included hyperlipidemia, osteoporosis, and hypothyroidism. On examination, motor power was intact (MRC grade 5/5) in bilateral lower limbs, except left ankle dorsiflexors: 4/5 and toe extensors: 3/5 with reduced sensation in L2 to L4 dermatomes bilaterally. There was no bladder or bowel incontinence. Plain radiographs revealed degenerative lumbar scoliosis with apex at L2 and left convexity, there was a lateral translation at L2–3 and L4–5 anterolisthesis (Fig. 6). The magnitude of the coronal deformity was 16.2° and lumbar lordosis was 35.3°. The patient also had a positive sagittal balance (SVA: + 9 cm) and pelvic parameters include PI: 65°, PT: 24°, and SS: 30.6°. Magnetic Resonance imaging revealed significant stenosis at L2–3 (Schizas Grade C) and L4–5 (Schizas Grade D) levels. In the first stage, she underwent L1–L4 OLIF. At L4–5 the surgical corridor was altered due to aberrant vascular anatomy. Hence at the second stage, L4–5 MIS TLIF with L1–L5 MIS pedicle screw instrumentation was performed. Postoperatively, lumbar lordosis improved to 55.2°, the PI-LL was 9.8°, and sagittal balance was restored (Fig. 7). At a 7-year follow-up, CT scan showed a solid fusion of the construct and a significantly improved VAS score (back pain).

## 5. Discussion

Adult denovo scoliosis is a complex condition primarily arising due to the progressive asymmetric degeneration of the discs and facet joints. As disc degeneration is a continuous process, the natural history aggravates the deformity resulting in a vicious cycle [5]. In patients with ASD, spinal instability, collapse in disc height, and flaval hypertrophy would contribute to features such as axial back pain, foraminal stenosis, and lateral recess stenosis. Various treatment options exist in the literature, ranging from decompression alone, in situ fixation, osteotomy with deformity correction, and long segment fixation. Though spinal canal decompression alone could alleviate the clinical symptoms of claudication, the deformity would continue to progress with associated instability and worsening back pain, especially in deformities of higher magnitude and spinal imbalance [6]. On the other hand, attempts at deformity correction could result in instrumentation spanning multiple motion segments, sometimes extending to the upper/mid-thoracic region. Traditionally, the posterior approach has been the major workhorse in patients with denovo scoliosis. Using the posterior-only approach, the fusion of long segments could result in increased stress on the spinal implants as the TLIF/PLIF cages have a relatively smaller footprint to support the anterior column [7]. In addition to other patient-related factors, this could result in surgical complications like pseudoarthrosis, rod breakage, and screw loosening as reported in the literature [8].

Hence the goal of surgery should not only be deformity correction and neural decompression but also at reducing implant-related post-operative adverse events. Recently, OLIF has emerged as one of the important Minimally invasive surgery strategies in treating these adult deformities, mainly as they provide better anterior column support using large cages and also aid in indirect decompression of the neural elements [9]. Studies have shown that the spinal canal dimensions improve significantly following OLIF and also the rate of pseudoarthrosis is reduced [10]. In our study, we have performed a two-staged approach (stage I: OLIF, stage II: posterior instrumentation) in the management of ASD, and by staging the surgery, the need for extensive hardware is reduced because the correction can be staged and segmented. This approach has several advantages, including: (1) reducing the problems associated with prolonged anesthesia, particularly in the elderly population; (2) allowing the surgeon to determine the pelvic parameters after the first surgery, which aids in deciding whether additional osteotomies or bone resections are needed during the second procedure, thereby minimizing the extent of posterior instrumentation and reducing the overall surgical morbidity; (3) providing the surgeon with guidance on the need for direct decompression at selected levels, in addition to an indirect decompression; and (4) offering the option of a completely minimally invasive approach using percutaneous pedicle screws without the need for posterior fusion. Thus the staged approach allows surgeons to tailor the posterior instrumentation and deformity correction based on the outcomes of the first stage. This customization can help in precisely addressing the deformity with optimal use of implants, avoiding overuse of instrumentation.

In our study, we observed that, in all three cases there was a significant correction of the coronal and sagittal imbalance. By spanning the cage along the long axis of the vertebral body, the asymmetric collapse of the disc could be effectively corrected. Also, in most patients with ASD, the lateral osteophytes would be one of the factors to affect effective correction of the deformity using a posterior approach. However, in OLIF, the lateral osteophytes could be effectively removed intra-operatively, improving the flexibility of the curve and aiding in improved correction of the deformity in the coronal plane. In one of our illustrated patients (case 2) with truncal translation, the coronal imbalance was about 10 cm, and post-OLIF, the correction achieved was more than 50 %, and at final post-operative radiographs, the patient was well-balanced in the coronal plane. Though initially, the plan was to extend the posterior construct to the upper thoracic level, following OLIF, as the stable vertebra was altered to a more distal level, a reduction in the length of the pedicle screw construct was possible.

In two cases (1 and 3), we were able to perform adequate decompression and deformity correction using an all-MIS approach (OLIF and percutaneous pedicle screw fixation ± tubular decompression). In both cases, there was a coronal imbalance, but after stage I OLIF, the coronal translation around the apex of the curve improved, the apical tilt was corrected and lumbar lordosis was established using the lordotic cages. Hence, the spanning of the construct was minimized only to the lumbar levels without extending into the thoracic spine. The other case (case 2) had a significant truncal transition along with a kyphosis at the thoracolumbar region. Hence, following the OLIF procedure, an open posterior approach spanning the instrumentation to T10 was performed with multiple posterior column osteotomies in the lumbar spine. It is well known that in ASD, sagittal imbalance is associated with a poor Health Related Quality Of Life (HRQOL) index [11,12]. In a recent meta-analysis, it was reported that OLIF combined with posterior column osteotomy (PCO) was superior to Pedicle Subtraction Osteotomy (PSO) in terms of SVA correction in degenerative spinal deformity. They noted a postoperative SVA mean correction of about 20 cm using lateral cages and PCO [13]. In our study, the sagittal balance was well maintained in all three cases, and by our two staged surgical (lateral and posterior) strategies; we believe we could provide an effective biplanar correction (coronal and sagittal) in ASD compared to only the posterior approach.

The incidence of peri-operative complications in ASD is quite high and Daubs et al. showed that in patients undergoing osteotomies for spinal deformity, the risk of major complications in patients over 69 years of age was ninefold more than the patients aged less than 69 years [14]. Therefore, the surgical treatment of ASD should be as minimally invasive as possible to reduce the risk of complications. With the advent of OLIF, the possibility of an all-minimally invasive option in such degenerative deformities has been established as it is a muscle-splitting approach with lesser soft tissue damage. Studies have reported an improvement in the overall radiological and clinical outcomes of ASD [15,16]. Radiologically, the lumbar lordosis and pelvic parameters could be effectively altered using OLIF and studies have shown that, in comparison to other MIS-fusions, OLIF in particular has a potential advantage in providing significant segmental lumbar lordosis [17]. This is possible because of the use of larger lordotic cages and by strategic placement of these cages anteriorly in the disc spaces. However, at the L5–S1 level, OLIF would not be possible in all cases due to anatomical constraints and we would recommend a lateral ALIF or MIS TLIF procedure at these levels.

Our study has the following limitations. This is a retrospective study with no control group. A prospective randomized study with a larger patient sample would be required to have a better understanding of other factors like proximal junctional failure/kyphosis. Patients factors like bone mineral density, and muscle mass were not evaluated in our study. Surgical data like blood loss and duration of surgery were not validated in our study. Also, the staging of the procedure could also add to the cost of the overall treatment of these patients. However, we believe that by using the circumferential MIS strategy, early patient recovery would be possible, reducing the duration of hospital stay and early return to work, thereby helping in the reduction of medical expenditure in the long term.

A staged approach in ASD (lateral-OLIF and posterior) is a valuable surgical strategy for patients with ASD. In addition, to the added advantage of staging the patient which allows the surgeon to reevaluate the extent of surgical instrumentation, it also helps to understand if the indirect decompression is effective. This could potentially be effective in reducing the overall complications in treating these pathologies. However, patient factors like osteoporosis, sarcopenia, and associated comorbidities must be considered carefully and a holistic approach would aid in improving the overall quality of life in these patients.
